# Placenta Prævia—Its Treatment

**Published:** 1878-06-20

**Authors:** Chas. F. A. Nichell

**Affiliations:** St. Helena, Cal.


### PLACENTA PRÆVIA—ITS TREATMENT.

CHAS. F. A. NICHELL, M.D., St. Helena, Cal.

The abnormal insertion of the placenta, known as placenta-prævia, has, since its recognition, always been regarded as one of the most dangerous complications o child bed; not only on account of the danger arising therefrom to the mother, but also from that to the child. Its importance, therefore, demands that all the light should be thrown upon it that clinical observation and experience may have gathered, and to direct the attention to each method of treatment that has secured the greatest safety to those intrusted to our care. With this aim in view, assured that the readers of your journal are too well acquainted with the etiology, diagnosis and prognosis of the subject under consideration for the writer to allude to them especially, he would submit the result of his observations while engaged in clinical work in Berlin.

The treatment of placenta-prævia depends *primarily* upon whether the hemorrhage occurs during the earlier period of gestation or near the close of the same, and *secondly*, upon the amount.

Hemorrhage occurring during the earlier period of gestation, and moderate in character (the first as a rule being slight), it will suffice to remand the patient to bed, placing her upon cool drinks, light and easily digested food, and securing free evacuation of the bowels by saline cathartics. Should the bleeding be arrested by these measures, the patient may be allowed to leave her bed after a few days, being, however, strictly charged to seek medicinal aid upon the first recurrence of the flow. Cold applications to the abdomen, vaginal injections, and the tampon are counter-indicated as these have a tendency to cause uterine contractions, thereby increasing the difficulty. Should the hemorrhage be ushered in, profuse and dangerous in character, the action of the physician will depend upon the condition of the cervix. When undilated so as to preclude the possibility of passing at least one finger beyond the os internum, he must have recourse to the tampon, compressing the bleeding surface between it and the ovum. Usually the bleeding will be arrested, but it will be prudent so long as no labor-pains are produced to continue the tampon for 3 or 4 days, changing it every 12 to 24 hours, according to the amount and character of the vaginal secretion. But should the hemorrhage continue, and the cervix be found impassable to one or two fingers, it will be necessary to dilate the same either with the sponge-tent or Barnes' dilators.

For the further operative interference intended by the physician, he must have a clear understanding of the character of the placental insertion, whether the same be *centralis* or *lateralis*, as upon a correct diagnosis, the prognosis of his case will largely depend. This, as at first sight might appear, is not always easy, since blood-coagula may be confounded with placenta.

In *placenta-prævia lateralis*, it will generally be sufficient, after the cervix has been made passable and the *head presenting*, to rupture the membranes. The hemorrhage will, as a rule cease at once, since the placenta can now follow as the uterus retracts over the advancing fœtal head, while the latter directly compresses the bleeding surface. This compression can be assisted by steady, firm pressure upon the uterine contents through the abdomen. With the discharge of the amniotic fluid, labor pains are ushered in, which increase in strength and regularity, and labor is completed, as a rule, without further accident. In case, however, the pains cease, and the hemorrhage arises anew, we must at once apply the forceps, providing always that the conditions requisite to their application are fulfilled, otherwise the delivery to be conducted in the manner subsequently considered.

In placenta-prævia lateralis should the head not present, version should be made by the combined (Braxton Hill) method. This method is also to be employed in *placenta-prœvia centralis* at whatever period of gestation the hemorrhage should occur, and irrespective of the fœtal presentation. As an especial advantage of this method, it must be borne in mind that, by bringing down a foot, the leg will act as a tampon from above downward, and as the cervix dilates we have a handle at our command by which we can at will increase the compressing power, by engaging the hip. Furthermore, by the early execution of the combined inner and outer version, we in a great measure avoid the anæmia as well as the danger from septic poisoning, which, notwithstanding the antiseptic precautions, we are likely to incur with the tampon. Since placenta prævia occurs usually in multipara in whom, during the last months of pregnancy, the cervix is passable for two fingers, the version with rupture of the membranes and bringing down a foot will be attended with but comparatively little difficulty. Certainly such a plan of operation is much easier and attended with far less danger than the accouchement force, by which lesions of the cervix occur, giving rise to most uncontrollable hemorrhages in the post-partum periods.

In the execution of the combined version for placenta-prævia, it will be necessary to attend to the following preliminary preparations :

*a.* Since it is important to operate as quickly as possible, it is imperative to entirely neutralize the action of the abdominal muscles. Accordingly, wherever practicable, we should chloroform the patient. This can be done with perfect safety notwithstanding a high grade of anæmia may exist.

*b.* The patient should be bedded, so as to allow the greatest freedom of action to tee operator. Care, however, should be had to maintain the horizontal position so as to guard against anæmia of the brain.

*c.* To avoid unnecessary delay in finding the feet, the position of the fœtus in utero must have been exactly determined by external palpation, before the operation begins.

The patient being thus prepared, we endeavor to pass the placenta. Unless we can readily feel the marginal border, or determine the smaller lobe of placenta, we proceed at once by a see-saw motion of the fingers to detach the placenta on that side, in which by our external examination we have found the feet to be, and as soon as we have reached the membranes rupture them. The fingers then advance in the direction of the feet, while the presenting part of the fœtus is displaced by the external manipulation, but not until the version is completed must we withdraw the hand, and with it bring down the foot. The leg or hip is to be brought down as far as the dilated os int. will permit. with which act the object of the operation, viz.: arrest of the hemorrhage, will have been accomplished. The further expulsion we leave to be accomplished by the labor-pains which incited by the foregoing operation will generally be found efficient. Should the cervix be not sufficiently dilated to permit the passage of, and offer great resistance to, the descent of the larger fœtal parts, steady but gentle traction upon the foot sufficient to compress the bleeding surface will answer the purpose, and the anæsthetic is to be discontinued. Care must be exercised in delivering the head, as frequently the os internum is not sufficiently dilated to permit its passage. Undue force to pass the fingers beyond the os internum will result in serious lesion, and must be guarded against. In such cases we must aim to engage the smallest diameter (bitemporal), which we may do by placing the middle finger upon the root of the tongue, the index and ring fingers upon the canine fossa, depressing the chin upon the chest, and by steady but gentle traction, assisted by external pressure, deliver the head. Under no circumstances must premature respiration on part of the fœtus lead us to a hasty delivery, as the danger arising therefrom to the child must give way to the greater, which the mother incurs by so rapid a delivery.

More or less profuse hemorrhage follows the expulsion of the head, even though no lesions of the cervix exist. This is due to the imperfect uterine contractions as they are especially met with in placenta prævia. The placenta if not readily expelled by the Crede method should at once be separated by the fingers.

But with the expulsion of the fœtus and placenta, the danger from hemorrhage to the mother is by no means past. For although by the combined inner and outer version we avoid laceration to the cervix, and the fœtal extremities compress the bleeding surface, yet owing to irregular uterine contractions, a large quantity of blood may discharge itself either externally or into the uterine cavity. Should a steady flow of blood exist, when the uterus is well contracted, it undoubtedly proceeds from lesions to the cervix. A recognition of the cause of the hemorrhage is of vital importance. When due to atony of the womb, friction and compression of the same, ergotin subcutaneously; ice-water and styptic injections into its cavity, but especially irrigation with water at a temperature of 122° to 125° F. will succeed in even the most desperate cases to secure contractions. Hemorrhage due to laceration of the cervix will render the foregoing measures futile, and our hope must depend upon direct compression. The syncope can be combatted with stimulants, hypodermic injections of tinct. musk, camphor, but especially by sulph. æther 15 to 20 minims repeated at short intervals.

Finally the after treatment will engage our attention in two directions. First, to secure an energetic involution, and, secondly, to overcome the anæmia. The former will be accomplished by occasional small doses of ergot, astringent vaginal injections, and hip-baths; the latter by horizontal posture with hips elevated, bandaging the lower extremities so as to force the blood to the heart, and such other measures as experience has sanctioned. However, should our efforts not be crowned with speedy success, the question of transfusion forces itself strongly upon our attention, and we should not defer this operation too long.—*Buffalo Med. Jour.*

